# Real-Time Synthetic Aperture Radar Imaging with Random Sampling Employing Scattered Power Mapping

**DOI:** 10.3390/s24123849

**Published:** 2024-06-14

**Authors:** Romina Kazemivala, Natalia K. Nikolova

**Affiliations:** Department of Electrical and Computer Engineering, McMaster University, Hamilton, ON L8S 4L8, Canada; talia@mcmaster.ca

**Keywords:** synthetic aperture radar, nonuniform sampling, imaging in real time, quantitative imaging, electromagnetic scattering, microwave imaging, millimeter-wave imaging, inverse scattering, nondestructive testing (NDT)

## Abstract

A novel image-reconstruction method is proposed for the processing of data acquired at random spatial positions. The images are reconstructed and updated in real time concurrently with the measurements to produce an evolving image, the quality of which is continuously improving and converging as the number of data points increases with the stream of additional measurements. It is shown that the images converge to those obtained with data acquired on a uniformly sampled surface, where the sampling density satisfies the Nyquist limit. The image reconstruction employs a new formulation of the method of scattered power mapping (SPM), which first maps the data into a three-dimensional (3D) preliminary image of the target on a uniform spatial grid, followed by fast Fourier space image deconvolution that provides the high-quality 3D image.

## 1. Introduction

In recent years, many microwave and millimeter-wave (mm-wave) imaging technologies have emerged to cater to a multitude of applications based on the principles of synthetic aperture radar (SAR). These innovative applications span various domains such as nondestructive testing and evaluation (NDT&E) [1,2,3,4,5,6,7], security inspection [8,9,10,11,12,13], and medical imaging [14,15,16,17,18,19,20]. Revolutionizing aerial surveillance, small unmanned aerial vehicles (UAVs) emerge as pivotal players in SAR imaging [21,22,23,24,25,26]. Their potential as platforms for SAR imaging is due to the remarkable progress of the positioning technology, which now provides accurate platform coordinates. The convergence of UAVs with ground-penetrating radars (GPRs) [27,28,29,30,31,32,33,34], operating at wavelengths as short as 73 mm, necessitates positional accuracy at the centimeter or millimeter scale for effective imaging. This accuracy, akin to trajectory accuracy in remote sensing, is of paramount importance since errors as small as a fraction of a wavelength lead to pronounced image distortions. Positional accuracy remains a challenge in handheld mm-wave imagers, where state-of-the-art systems rely on fast electronically switched antenna arrays forming the acquisition aperture [35,36,37]. Nonetheless, handheld imagers stand to benefit greatly from the addition of positioning technology since this can dramatically expand the realized synthetic aperture beyond the size of the switched antenna array. A common requirement for the image-reconstruction algorithms of such UAV-borne or handheld imaging systems is that they must be able to process randomly sampled datasets acquired in three-dimensional (3D) space with coordinates which do not conform to uniform grids.

In conventional close-range imaging, mechanical scanning is typically realized with 1D or 2D automated scanners (also known as translation stages), equipped with high-precision positioning mechanisms. The sampling is usually uniform, i.e., at regular spatial intervals, although advanced scanners also allow for random sampling. The scanning, however, is inherently constrained to canonical (planar, cylindrical, and spherical) surfaces. Most of the real-time image-reconstruction algorithms (e.g., microwave holography and the range migration) take advantage of such uniformly sampled data in order to employ fast Fourier transform (FFT) algorithms and to achieve remarkable reconstruction speeds [38,39,40]. However, this also renders them incapable of processing randomly sampled data. Nonuniform sampling on canonical acquisition surfaces can be handled by interpolation, but this entails significant computational overhead and may result in unfocused images [41,42,43]. On the other hand, nonuniform sampling on such surfaces has been shown to allow for sampling densities below the Nyquist limit [44,45,46].

In summary, innovative solutions are needed to address nonuniform and random sampling since they would benefit both the imaging systems sampling along unpredictable 3D trajectories (e.g., handheld and UAV-borne) and those scanning over canonical 2D surfaces.

To this end, a few image-reconstruction algorithms based on the back-projection (or back-propagation) algorithm (BPA) have been proposed [24,27,29,47,48]. The BPAs, when applied in real (x,y,z) space, can inherently handle any sampling position; thus, they are preferred in the case of nonuniform and random sampling. However, the computational resources required by the BPAs are significant as they rely on aggregating phase-conjugated scattered field data [49,50]. This may preclude real-time image reconstruction. The accuracy of the sampling positions is critical in imaging reconstruction, and it is a major problem with moving SAR platforms. Various approaches to mitigate positional inaccuracies through hardware adjustments are employed [51,52,53] to make the data suitable for BPA processing. These methods employ an integrated navigation system to monitor the real-time motion velocity of the radar platform. Among the BPAs, some incorporate interpolation of the nonuniformly sampled signals [52,54]. Other BPAs incorporate image fusion to increase the quality of the images by splitting the large synthetic aperture into multiple sub-apertures [24,52]. Further, in order to accurately extract the velocity to obtain high-quality BPA images, the adaptive notch filtering (ANF) technique has been employed [47].

The ω-*k* algorithms, such as the range-migration algorithms (RMAs) and the microwave holography algorithms (MHAs), provide a compelling alternative to the BPAs [42,49,55,56,57,58]. These methods first transform the data sampled over the aperture into the Fourier spatial-frequency domain (the *k*-space) through FFT. These algorithms harness an analytical range-migration model, which links the frequency (ω) dependence of the data to the range (or depth) position of a scattering center within the imaged object. This is facilitated by Stolt’s interpolation. The object’s reflectivity is reconstructed in *k*-space, after which it is translated back into real space via the inverse Fourier transform (IFT). By operating mostly in *k*-space, the ω-*k* algorithms gain a significant speed advantage over the BPAs. However, they are limited to uniform sampling on canonical surfaces. This limitation is overcome with various methods referred to as spectral estimation [56]. The spectral estimation aims at recovering the spectrum of the data on a uniform grid in *k*-space while the data are acquired with nonuniform sampling on the synthetic aperture (in real space). Methods include natural neighbor interpolation, nonuniform FFT (NUFFT), nonuniform discrete Fourier transform (NDFT), and multilevel conjugate-gradient residual error minimization. The spectral estimation comes at a significant computational cost. Moreover, it remains limited by the requirement for a synthetic aperture of canonical shape.

Various recent studies focus on data preprocessing for randomly and nonuniformly sampled data rather than new image-reconstruction algorithms. The respective approaches can be categorized into regularization-based algorithms [59,60,61,62,63,64] and compressive-sensing (CS) algorithms [65,66,67,68,69,70,71,72]. The latter can address not only the random sampling but can also potentially deal with data sparsity, thus reducing the required amount of measurements [67]. As an example of a regularization-based algorithm, Zhang et al. [59] formulate a linear system of equations, where the system matrix comprises Fourier coefficients. Tikhonov regularization is employed to solve the system of equations, providing the 1D *k*-spectrum of the nonuniform spatially sampled data along the azimuth. The application is geared toward linear frequency-modulated (LFM) radar signals. In effect, the preprocessing performs spectral estimation before providing the *k*-space result to an image-reconstruction algorithm. Uniformly sampled *k*-space data can be processed with various *k*-space image-reconstruction algorithms such as the RMAs, the MHAs, or the range Doppler algorithm (RDA) [59,60]. On the other hand, the CS techniques are primarily focused on achieving high image resolution with sparse data, where the sampling rate is well below the Nyquist limit [65,66]. The sparse data may be uniformly or randomly sampled, but there seems to be benefits in the latter case [67,73]. The main limitation of CS is that the outcome strongly depends on the adequate choice of the representation basis [67], where the information content of the data is anticipated to be much lower than in the actual measurement space [73]. In imaging, the existence of such basis and the data sparsity in it are dictated by the complexity of the imaged object and some distinct shape or material features. Therefore, CS algorithms do not compensate for the lack of data; they leverage mathematical representations of signals known to be presentable in terms of only a few basis functions in some space [74].

Scattered power mapping (SPM) is a well-established method for rapid (real-time) SAR imaging, which has been applied with both frequency-domain (stepped frequency continuous wave, SFCW) and LFM data [38,75,76,77,78]. This paper proposes a new SPM algorithm adapted to perform image reconstruction with nonuniformly sampled data acquired on random 3D trajectories—a scenario arising when imaging radars are mounted on mobile or handheld platforms. We emphasize that the proposed method is not a compressed sensing approach. The hallmark of SPM is a two-stage inversion, where the first stage is a projection that maps the data onto a 3D image of the target reflectivity, and the second stage is an image deconvolution which enhances the output of the first stage. The first (projection) stage of the algorithm can work either in real (x,y,z) space [75,76] or in *k*-space [77,78]. The *k*-space implementation is computationally very efficient, but this efficiency relies on the FFT of the data, which in turn requires uniform sampling on canonical synthetic apertures. In contrast, the real-space implementation can handle any sampling position while still producing a reflectivity image on a uniform 3D grid. Therefore, it is capable of utilizing data that are randomly sampled in 3D space. On the other hand, the second (image-deconvolution) stage is always performed in *k*-space since it operates on the reflectivity output of the first stage, which is already cast onto a uniform 3D grid. Thus, the second stage does not depend on whether the sampling is random or uniform, and it is very fast.

In summary, the proposed image reconstruction has the flexibility of BPAs to handle randomly sampled data, but it yields images of higher quality due to the image-deconvolution stage. In comparison with the ω-*k* algorithms, the proposed algorithm does not require synthetic apertures on canonical surfaces. The randomly sampled aperture is in fact a 3D volume. Neither NUFFT nor other spectral estimation methods are needed, which renders the reconstruction faster than that with the ω-*k* algorithms.

Finally, the algorithm processes the data on-the-run, i.e., the image reconstruction does not need to wait for the completion of a scan. In fact, utilizing a convergence criterion, it is the algorithm which decides when the amount of data is sufficient and its collection can be terminated.

## 2. Methodology

### 2.1. 3D Scanning

**Figure 1:**
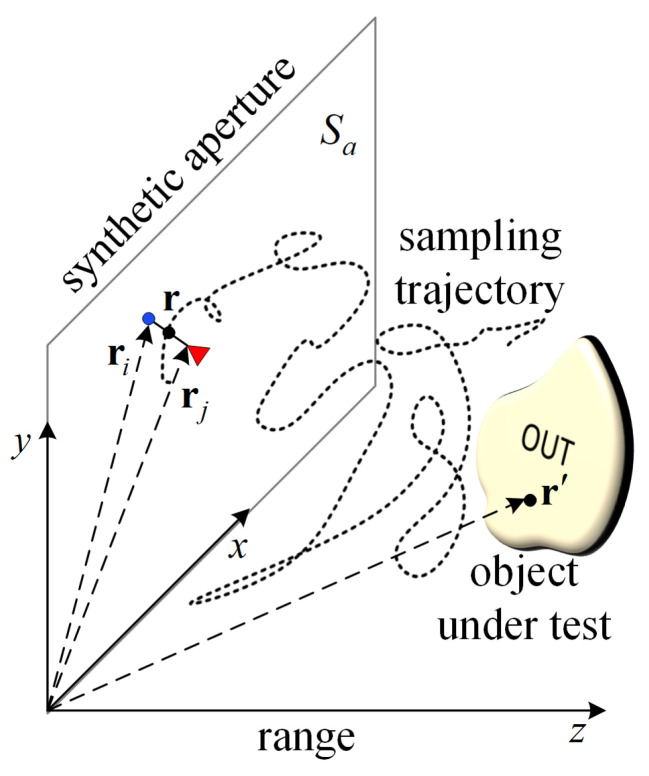
Illustration of a measurement on a random 3D trajectory (black dots) as opposed to a measurement on a planar aperture denoted as $S_{a}$. The trajectory may also be 2D, lying entirely within $S_{a}$. The red triangle and the blue point represent Tx and Rx positions, respectively. The response acquired with the *i*-th Rx antenna ($i=1,\ldots ,N_{\mathrm{Rx}}$) and the *j*-th Tx antenna ($j=1,\ldots ,N_{\mathrm{Tx}}$) is identified by a subscript ζ≡(i,j). The platform’s position r is assumed known at each measurement instance, and the positions of the Rx and Tx antennas, $r_{i}$ and $r_{j}$, respectively, are determined from r. An imaged position is denoted as $r^{\prime }$. Note that the bore–sight axis of the antennas is fixed along the range direction.

Figure 1 illustrates a scan along a random 3D trajectory as opposed to a scan on a planar 2D synthetic aperture. The positions of the imaging platform along the trajectory, where measurements are taken, are assumed known and are denoted as r. These positions are illustrated by the dots comprising the trajectory. Each measurement instance is associated with $N_{\mathrm{Tx}}$ transmitting (Tx) antennas and $N_{\mathrm{Rx}}$ receiving (Rx) antenna positions to allow for the possibility of utilizing a multiple-input multiple-output (MIMO) radar. The Tx and Rx antennas are rigidly mounted on the platform; therefore, it is assumed that the Rx antenna positions $r_{i}$ ($i=1,\ldots ,N_{\mathrm{Rx}}$) and the Tx antenna positions $r_{j}$ ($j=1,\ldots ,N_{\mathrm{Tx}}$) can be determined from the platform’s position r. An imaged position is denoted by $r^{\prime }$. The measurement may be carried out using SFCW or LFM radars, examples of which are presented here. The SPM algorithm can also be applied with pulsed radar data. Using multiple antennas on a moving platform has several advantages. Firstly, multiple Tx and Rx antennas offer diversity in illumination and observation angles, respectively, which is beneficial in close-range measurements. Secondly, having more data from multiple antennas significantly reduces image noise and clutter [79]. Lastly, these benefits drive the development of MIMO radars, where the array element spacing is about half a wavelength or more, enhancing phase diversity in collected responses. Note that in our investigation, the tilt angle of the antenna relative to the range is constant. Thus, the platform’s orientation does not vary. Furthermore, the antenna far-field patterns are not considered in the presented examples. The use of antenna patterns in SAR imaging is well-known, and its benefits have been investigated in [80,81]. While it can enhance accuracy, its use is often impractical due to the unavailability of 3D patterns or near-zone measurement conditions [3,6,40,44,63,77,82,83,84,85].

### 2.2. Image Reconstruction with Randomly Sampled Data Employing Scattered Power Mapping

The SPM is a two-stage inversion method for real-time SAR imaging [75,76,77,78]. It has been originally applied with frequency-domain data, for which an exact forward model of scattering exists in terms of the *S*-parameters [86]. For real-time image reconstruction, this exact model is linearized using the zero-order Born approximation of the total internal field [38], leading to the approximate data equation for the scattered portion of the measured *S*-parameters:

(1) $$S_{\zeta }^{\mathrm{sc}}\left(r,\omega \right)\approx c_{\zeta }{\;\int \int \int \;}_{V^{\prime }}\Delta \varepsilon_{r}\left(r^{\prime }\right)E_{\zeta ,\mathrm{Rx}}^{\mathrm{inc}}\left(r^{\prime },r,\omega \right)\cdot E_{\zeta ,\mathrm{Tx}}^{\mathrm{inc}}\left(r^{\prime },r,\omega \right)dr^{\prime }\;.$$

Here, ω is frequency, r is the measurement position, $r^{\prime }\in V^{\prime }$ is a position within the imaged volume $V^{\prime }$, and ζ≡(i,j) denotes the antenna pair determined by the Rx (*i*-th) and Tx (*j*-th) antennas. For instance, $\zeta =1,2,3,\ldots ,N_{\mathrm{Tx}}N_{\mathrm{Rx}}$ refers to $S_{11},S_{22},S_{21},$ etc. Here, $E_{\zeta ,\mathrm{Tx}}^{\mathrm{inc}}$ is the incident field due to the Tx antenna at $r_{j}\left(r\right)$, which replaces the total internal field through Born’s zero-order approximation, and $E_{\zeta ,\mathrm{Rx}}^{\mathrm{inc}}$ is the incident field due to the Rx antenna at $r_{i}\left(r\right)$, if this antenna were to transmit. Mathematically, $E_{\zeta ,\mathrm{Rx}}^{\mathrm{inc}}$ represents the background vector Green function. Additionally, $\Delta \varepsilon_{r}\left(r^{\prime }\right)$ is the object’s relative-permittivity contrast, which is assumed independent of the frequency and the field polarization. The object’s contrast is in general complex, i.e., it can represent lossy materials. It is defined as $\Delta \varepsilon_{r}=\varepsilon_{r}-\varepsilon_{r,b}$, where $\varepsilon_{r}$ is the object’s complex relative permittivity and $\varepsilon_{r,b}$ is that of the background. The constant $c_{\zeta }=\frac{-i\omega \varepsilon_{0}}{2a_{\mathrm{Rx}}a_{\mathrm{Tx}}}$ is determined by the root-power waves $a_{\mathrm{Rx}}$ and $a_{\mathrm{Tx}}$ (root-power waves, or power waves, are phasors that describe traveling electromagnetic waves in waveguides [38,87]. The magnitude is the square root of the power carried by the wave, and the phase corresponds to the phase of the wave’s electric field) and the incident on the ports of the antennas generating the respective fields. Here, $\varepsilon_{0}$ denotes the permittivity of the vacuum.

Here we assume that the objects are nonmagnetic and that the scattering is polarization-independent. Additionally, we assume that the permittivity is constant in the frequency band of interest (the object is nondispersive) [88,89,90] since dispersion effects are weak at microwave and millimeter-wave frequencies. However, the E-fields and the *S*-parameters (the data) in (1) are frequency-dependent, as dictated by the wavenumbers of the background and the object as well as the signal phase delays to/from the object. This frequency dependence provides response diversity and is critical in achieving high image quality.

For the purposes of the discussion that follows, the forward model of scattering must be cast in terms of the system PSF, which plays a central role in the proposed SPM method. The system PSF represents the response (the measured signal) due to a point scatterer in the background medium [38]. It is clear that the PSF of the data Equation (1) is

(2) $$H_{\zeta }^{\mathrm{sc}}\left(r,\omega ;r_{\mathrm{sp}}\right)=c_{\zeta }\Omega_{\mathrm{sp}}\Delta \varepsilon_{r,\mathrm{sp}}E_{\zeta ,\mathrm{Rx}}^{\mathrm{inc}}\left(r_{\mathrm{sp}},r,\omega \right)\cdot E_{\zeta ,\mathrm{Tx}}^{\mathrm{inc}}\left(r_{\mathrm{sp}},r_{\mathrm{Tx}},\omega \right)\;,$$

describing the scattering from an electrically small (point-like) scatterer of volume $\Omega_{\mathrm{sp}}$ and contrast distribution represented by Dirac’s δ-function, $\Delta \varepsilon_{r,\mathrm{sp}}\delta \left(r^{\prime }-r_{\mathrm{sp}}\right)$. Here, $r_{\mathrm{sp}}$ is the position of the point scatterer (also referred to as the scattering probe). The system-specific kernel in (2) can be determined through a calibration measurement independently of the OUT. By measuring an electrically small scatterer of known $\Omega_{\mathrm{sp}}$ and $\Delta \varepsilon_{r,\mathrm{sp}}$ embedded in the background medium, one can obtain the measured PSFs $H_{\zeta }^{\mathrm{sc}}\left(r,\omega ;r_{\mathrm{sp}}\right)$. In close-range radar imaging applications, measuring the PSF with a high SNR is feasible as long as the scattering probe can be embedded in the background medium. In long-range radar imaging, measuring the PSF is challenging since the scattering signal from a distant small probe is very weak, thus the preference for using analytical PSFs.

As suggested by (1), acquiring the incident fields distribution as represented by the dot product is complex. If the PSF is acquired as shown in (2), there is a simple linear relation between (1) and (2). The linearized scattering model (1) is then expressed as

(3) $$S_{\zeta }^{\mathrm{sc}}\left(r,\omega \right)\approx {\;\int \int \int \;}_{V^{\prime }}\rho \left(r^{\prime }\right)H_{\zeta }^{\mathrm{sc}}\left(r,\omega ;r^{\prime }\right)dr^{\prime }\;,$$

where $\rho \left(r^{\prime }\right)=\Delta \varepsilon_{r}\left(r^{\prime }\right)/\left(\Delta \varepsilon_{r,\mathrm{sp}}\Omega_{\mathrm{sp}}\right)$ is termed the object’s reflectivity function. Clearly, the model in (3) views the imaged object as a collection of point (or differential) scatterers and represents the measured signal as the superposition of the scattering emanating from all point scatterers making up the object. Note that the relation between the measured PSF and the incident fields due to the Tx and Rx antennas are determined by comparing (1) and (3). Further, (2) is crucial in computing quantitative estimations of permittivity. The advantage of the representation in (3) is that it allows for using a PSF, which is derived either analytically, or from simulations, or from measurements. The analytical PSFs typically employ far-field approximations of the incident fields in (2) (possibly enhanced by the antenna’s far-field patterns [81]) whereas simulation-based PSFs utilize incident field distributions obtained by simulating the radiation from the Rx and Tx antennas in the background medium [91]. Measured PSFs are obtained by measuring an electrically small scattering probe of known volume $\Omega_{\mathrm{sp}}$ and contrast $\Delta \varepsilon_{r,\mathrm{sp}}$ placed at the center of the imaged volume [75,92].

With the data $S_{\zeta }^{\mathrm{sc}}\left(r,\omega \right)$ and the PSFs $H_{\zeta }^{\mathrm{sc}}\left(r,\omega ;r^{\prime }\right)$ available, the SPM reconstructs $\rho (r^{\prime })$ through a computationally efficient two-stage procedure. The first SPM stage is a projection that builds the 3D scattered power map $M(r^{\prime })$ of the object under test (OUT) as the discrete inner product of the data and the system PSFs:

(4) $$M\left(r^{\prime }\right)=\sum_{\zeta =1}^{N_{R}}\sum_{n=1}^{N_{\omega }}\sum_{m=1}^{N_{m}}S_{\zeta }^{\mathrm{sc}}\left(r_{m},\omega_{n}\right)\;{\left[H_{\zeta }^{\mathrm{sc}}\left(r_{m},\omega_{n};r^{\prime }\right)\right]}^{*}\;.$$

Here, $N_{m}$ is the number of measurement positions, $N_{\omega }$ is the number of frequency points, and $N_{R}$ is the total number of used antenna pairs (the number of responses) at each measurement position r and each frequency ω as determined by the available Tx/Rx antenna pairs in ζ=(i,j). The measurement positions $r_{m}$, $m=1,\ldots ,N_{m}$ may belong to a synthetic 2D aperture, a 3D acquisition volume, or a random trajectory. We emphasize that we do not consider the variation in the platform orientation and it is fixed along the antennas’ bore–sight axis. Note that the OUT power map $M(r^{\prime })$ has complex values. With a large number of responses, this map converges to a distribution, which is proportional to the OUT complex permittivity $\Delta \varepsilon_{r}\left(r^{\prime }\right)$ [38]. Therefore, $\left|M(r^{\prime })\right|$ is a representation of the OUT reflectivity $\left|\rho (r^{\prime })\right|$, and its normalized plot provides a qualitative OUT image.

Recently, it has been shown that the projection reconstruction stage described by (4) can also be applied with time-domain signals $S_{\zeta }^{\mathrm{sc}}\left(r,t\right)$[78], where the summation over ω is simply replaced by a summation over time *t*:

(5) $$M\left(r^{\prime }\right)=\sum_{\zeta =1}^{N_{R}}\sum_{n=1}^{N_{n}}\sum_{m=1}^{N_{m}}S_{\zeta }^{\mathrm{sc}}\left(r_{m},t_{n}\right)\;{\left[H_{\zeta }^{\mathrm{sc}}\left(r_{m},t_{n};r^{\prime }\right)\right]}^{*}\;.$$

Note that the conjugation of $H_{\zeta }^{\mathrm{sc}}\left(r,t;r^{\prime }\right)$ matters in the case of LFM radar signals, which are complex, i.e., at each r, the receiver output contains an in-phase (*I*) and a quadrature (*Q*) signal component so that the time-dependent signal is expressed as S(t)=I(t)+iQ(t).

The first SPM stage carried out with (4) for frequency-domain signals or with (5) for time-domain signals allows for mapping the random spatial samples (r) onto a uniform 3D image grid (r’). Moreover, since the OUT map $M(r^{\prime })$ is computed as the sum of contributions $C_{m}\left(r^{\prime }\right)$ from the measurement positions $r_{m}$ ($m=1,\ldots ,N_{m}$),

(6) $$M\left(r^{\prime }\right)=\sum_{m=1}^{N_{m}}C_{m}\left(r^{\prime }\right)\;,\;C_{m}\left(r^{\prime }\right)=\sum_{\zeta =1}^{N_{R}}\sum_{n=1}^{N_{n}}S_{\zeta }^{\mathrm{sc}}\left(r_{m},\nu_{n}\right)\;{\left[H_{\zeta }^{\mathrm{sc}}\left(r_{m},\nu_{n};r^{\prime }\right)\right]}^{*}\;,\;\nu =\omega \;\mathrm{or}\;t\;,$$

it can be updated concurrently with the measurements by adding a measurement contribution when it becomes available, assuming that the PSF $H_{\zeta }^{\mathrm{sc}}\left(r_{m},\nu_{n};r^{\prime }\right)$ has been determined on a uniform grid $r^{\prime }$ in the OUT power map for random measurement positions $r_{m}$. In (6), $N_{n}$ is the number of frequency or temporal samples. We emphasize that the method is versatile and not limited to specific frequency bands, as the contribution of each frequency/time sample $\nu_{n}$ is being summed as shown in (6). As the number of data points increases with the stream of additional measurements, this map converges to a first-stage OUT image, which is further processed by the second SPM stage. We reiterate that the imaged positions $r^{\prime }$ belong to a uniformly sampled spatial grid whereas the measurement (or observation) positions $r_{m}$, $m=1,\ldots ,N_{m}$ are random.

The second SPM stage performs image deconvolution, which greatly enhances the image quality. This deconvolution is performed efficiently in Fourier (*k*) space, taking advantage of the fact that the first-stage OUT map $M(r^{\prime })$ in (6) is already cast on a uniformly sampled grid. Assuming a homogeneous unbounded (an unbounded medium is a standard term in numerical electromagnetics indicating that the domain of interest is “open”, i.e., without exterior reflecting boundaries. In EM simulations, this is achieved using open-space Green’s functions or nonreflecting boundary conditions, e.g., perfectly matched layers) background, $M(r^{\prime })$, $r^{\prime }\equiv \left(x^{\prime },y^{\prime },z^{\prime }\right)$ can be expressed through the 2D convolution of the reflectivity function $\rho (x^{\prime },y^{\prime },z^{\prime })$ and the point-scatterer maps $M(x^{\prime },y^{\prime },z^{\prime };z^{\prime \prime })$ [38,75,77]:

(7) $$M\left(x^{\prime },y^{\prime },z^{\prime }\right)=\underset{z^{\prime \prime }y^{\prime \prime }x^{\prime \prime }}{\;\int \int \int \;}\rho \left(x^{\prime \prime },y^{\prime \prime },z^{\prime \prime }\right)\;M\left(x^{\prime }-x^{\prime \prime },y^{\prime }-y^{\prime \prime },z^{\prime },z^{\prime \prime }\right)dx^{\prime \prime }dy^{\prime \prime }dz^{\prime \prime }\;.$$

The second SPM stage aims at recovering the unknown reflectivity function $\rho (r^{\prime })$. The point-scatterer map $M(x^{\prime },y^{\prime },z^{\prime };z^{\prime \prime })$ is the first-stage map of a point scatterer residing at the center $\left(x^{\prime \prime }=y^{\prime \prime }=0\right)$ of the image slice at $z^{\prime \prime }$ in the homogeneous background. Hereafter, we refer to this map as a PSF map. Note that for each image slice $z^{\prime \prime }$, there is a corresponding 3D PSF map. The PSF map $M(x^{\prime },y^{\prime },z^{\prime };z^{\prime \prime })$ is defined analogously to the first-stage OUT projection (6), where the OUT response $S_{\zeta }^{\mathrm{sc}}\left(r_{m},\nu_{n}\right)$, $r_{m}=\left(x_{m},y_{m},z_{m}\right)$, is replaced by the PSF $H_{\zeta }^{\mathrm{sc}}\left(r_{m},\nu_{n};z^{\prime \prime }\right)$ representing the response from a scattering probe at $r^{\prime \prime }=\left(0,0,z^{\prime \prime }\right)$:

(8) $$\begin{array}{c} M\left(x^{\prime },y^{\prime },z^{\prime };z^{\prime \prime }\right)=\sum_{m=1}^{N_{m}}\sum_{\zeta =1}^{N_{R}}\sum_{n=1}^{N_{n}}H_{\zeta }^{\mathrm{sc}}\left(x_{m},y_{m},z_{m},\nu_{n};z^{\prime \prime }\right)\;\;\;\;\;\;\;\;\;\;\;\;\;\;\;\;\;\;\;\;\;\;\;\;\;\;\;\;\;\;\;\;\;\;\;\;\;\;\;\;\;\;\;\;\;\;\;\;\;\;\;\;\;\;\;\;\\ \;\;\;\;\;\;\;\;\;\;\;\;\;\;\;\;\;\;\;\;\;\;\;\;\;\;\;\;\;\;\;\;\;\;\;\;\;\;\;\;\;\;\;\;\;\;\;\;\;\;\;\;\;\;\;\;{\left[H_{\zeta }^{\mathrm{sc}}\left(x_{m},y_{m},z_{m},\nu_{n};x^{\prime },y^{\prime },z^{\prime }\right)\right]}^{*}\;,\;\nu =\omega \;\mathrm{or}\;t\;. \end{array}$$

In contrast to the OUT map, however, the PSF maps do not need to be computed from spatially random samples. The PSFs $H_{\zeta }^{\mathrm{sc}}$ are independent of the OUT and they can be obtained analytically, or via simulations, or via measurements, on any convenient grid of observation points r. From a computational efficiency point of view, a PSF evaluation on a uniform grid is advantageous since it allows for the deconvolution of (7) to be performed in *k*-space, as explained next.

We assume that the PSFs are evaluated on a planar synthetic aperture $S_{a}$ (see Figure 1), whose range position $\bar{z}$ relative to the OUT is chosen to be within the span of the range positions $z_{m}$, $m=1,\ldots ,N_{m}$ during the OUT random scan. The lateral rectangular extent of $S_{a}$ is also determined by the minimum and maximum lateral (*x* and *y*) coordinates of the random samples. The sampling along *x* and *y* on this plane is coincident with the grid points along $x^{\prime }$ and $y^{\prime }$ of the OUT map. This sampling must be sufficiently dense so that it conforms to the Nyquist limit. For example, with monostatic radar measurements, the spatial sampling steps Δx and Δy should not exceed $\frac{\lambda_{\min}}{4\sin\alpha }$[38], where α is the maximum viewing angle determined by the aperture size and the antenna beamwidths. The assumptions of a homogeneous background and uniform sampling along (x,y) allow for casting (8) in terms of a discrete 2D convolution:

(9) $$\begin{array}{c} M\left(x^{\prime },y^{\prime },z^{\prime };z^{\prime \prime }\right)=\sum_{\chi =1}^{N_{x}}\sum_{\eta =1}^{N_{y}}\sum_{\zeta =1}^{N_{R}}\sum_{n=1}^{N_{n}}H_{\zeta }^{\mathrm{sc}}\left(x_{\chi },y_{\eta },\bar{z},\nu_{n};z^{\prime \prime }\right)\;\;\;\;\;\;\;\;\;\;\;\;\;\;\;\;\;\;\;\;\;\;\;\;\;\;\;\;\;\;\;\;\;\;\;\;\;\;\;\;\;\;\;\;\;\;\;\;\;\;\;\;\;\;\;\;\\ \;\;\;\;\;\;\;\;\;\;\;\;\;\;\;\;\;\;\;\;\;\;\;\;\;\;\;\;\;\;\;\;\;\;\;\;\;\;\;\;\;\;\;\;\;\;\;\;\;\;\;\;\;\;\;\;{\left[H_{\zeta }^{\mathrm{sc}}\left(x_{\chi }-x^{\prime },y_{\eta }-y^{\prime },\bar{z},\nu_{n};z^{\prime }\right)\right]}^{*}\;,\;\nu =\omega \;\mathrm{or}\;t\;. \end{array}$$

In *k*-space, the 2D convolution in (9) is a multiplication of the 2D FTs of the PSF responses:

(10) $$\tilde{M}\left(k_{x},k_{y},z^{\prime };z^{\prime \prime }\right)=\sum_{\zeta =1}^{N_{R}}\sum_{n=1}^{N_{n}}{\tilde{H}}_{\zeta }^{\mathrm{sc}}\left(k_{x},k_{y},\bar{z},\nu_{n};z^{\prime \prime }\right){\left[{\tilde{H}}_{\zeta }^{\mathrm{sc}}\left(k_{x},k_{y},\bar{z},\nu_{n};z^{\prime }\right)\right]}^{*}\;,\;\nu =\omega \;\mathrm{or}\;t\;.$$

Here, $\tilde{M}\left(k_{x},k_{y},z^{\prime };z^{\prime \prime }\right)$ is the 2D FT of the PSF map $M(x^{\prime },y^{\prime },z^{\prime };z^{\prime \prime })$; ${\tilde{H}}_{\zeta }^{\mathrm{sc}}\left(k_{x},k_{y},\bar{z},\nu_{n};z_{\mathrm{sp}}\right)$ is the 2D FT of the PSF response $H_{\zeta }^{\mathrm{sc}}\left(x,y,\bar{z},\nu ;z_{\mathrm{sp}}\right)$ when the scattering probe is at the center of the range slice $z_{\mathrm{sp}}=z^{\prime },z^{\prime \prime }$; and $k_{x}$ and $k_{y}$ are the Fourier variables corresponding to $x^{\prime }$ and $y^{\prime }$, respectively. Although the computation in (10) requires the 2D FT of the PSFs, it is orders of magnitude faster than its real-space counterpart in (9) [77]. Moreover, it is the *k*-space PSF maps that are needed for the efficient deconvolution of (7) and the recovery of the reflectivity function $\rho (r^{\prime })$, as explained next.

Applying 2D FT to both sides of (7) and discretizing the integration over $z^{\prime \prime }$ into a sum leads to

(11) $$\tilde{M}\left(k_{x},k_{y},z_{p}^{\prime }\right)=\Delta \Omega_{v}\sum_{k=1}^{N_{z}}\tilde{\rho }\left(k_{x},k_{y},z_{k}^{\prime \prime }\right)\tilde{M}\left(k_{x},k_{y},z_{p}^{\prime };z_{k}^{\prime \prime }\right)\;,p=1,\ldots ,N_{z}\;,$$

where $N_{z}$ is the number of image slices, $\Delta \Omega_{v}=\Delta x\Delta y\Delta z$ is the image voxel volume, $\tilde{M}\left(k_{x},k_{y},z_{p}^{\prime }\right)$ is the 2D FT of $M(x^{\prime },y^{\prime },z_{p}^{\prime })$, and $\tilde{\rho }\left(k_{x},k_{y},z_{k}^{\prime \prime }\right)$ is the 2D FT of $\rho (x^{\prime \prime },y^{\prime \prime },z_{k}^{\prime \prime })$. The relation in (11) defines a square linear system of $N_{z}$ equations for the $N_{z}$ unknowns $\tilde{\rho }\left(k_{x},k_{y},z_{k}^{\prime }\right),\;k=1,\cdots ,N_{z}$ solved at each point in Fourier space $\left(k_{x},k_{y}\right)$. With the *k*-space reflectivity function found, the real-space reflectivity $\rho (x^{\prime },y^{\prime },z_{k}^{\prime })$ is recovered slice by slice via the inverse 2D FT:

(12) $$\rho \left(x^{\prime },y^{\prime },z_{n}^{\prime }\right)=F_{2D}^{-1}\left\{\tilde{\rho }\left(k_{x},k_{y},z_{k}^{\prime }\right)\right\}\;,k=1,\ldots ,N_{z}\;.$$

The plot of $\left|\rho (x^{\prime },y^{\prime },z_{n}^{\prime })\right|$, usually normalized, provides a qualitative image of the object’s reflectivity. A quantitative image is also possible, provided the system PSFs scale properly with the probe’s volume $\Omega_{\mathrm{sp}}$ and relative-permittivity contrast $\Delta \varepsilon_{r,\mathrm{sp}}$. The quantitative estimate of the object’s relative-permittivity contrast is obtained as

(13) $$\Delta \varepsilon_{r}\left(x^{\prime },y^{\prime },z_{n}^{\prime }\right)=\Omega_{\mathrm{sp}}\Delta \varepsilon_{r,\mathrm{sp}}\rho \left(x^{\prime },y^{\prime },z_{n}^{\prime }\right)\;.$$

### 2.3. Image-Convergence Check

The computation of the OUT power map $M(x^{\prime },y^{\prime },z^{\prime })$ (the first-stage SPM image) involves a summation over the contributions from all available measured positions $r_{m}$, $m=1,\ldots ,N_{m}$; see (6). However, unlike measurements with mechanical or electronically switched scanners, the number of spatial samples $N_{m}$ is not known a priori. Vehicle-borne or handheld imaging platforms usually cannot follow exactly a predetermined trajectory. In this case, the data collection must continue until a convergent image emerges such that the addition of more data points no longer contributes to its quality. Therefore, a criterion for image convergence is needed.

To assess the convergence of the evolving image, we utilize the structural similarity index measure (SSIM) between two consecutive OUT power maps after an incremental addition of random acquisition points. The SSIM varies between 0 (no similarity) and 1 (perfect match). Thus, an SSIM value above a certain threshold (0.97 or higher) indicates that adding more data points would no longer contribute to the image improvement and the scan can be terminated.

While the above concept is straightforward, it does not guarantee sufficient sampling by itself. The sampling must be distributed over a broad synthetic aperture to provide the widest possible range of viewing angles of the imaged object. This is critical for achieving the best possible image spatial resolution [38]. To this end, we first determine the lateral extent of the observation domain based on the size of the imaged object, the average stand-off distance, and the beamwidth of the used antennas. For example, the *x* axis extent of a planar synthetic aperture $X_{a}$ for an object of size $X_{o}$ is

(14) $$X_{a}=X_{o}+2\bar{z}\tan\left(0.5\theta_{\mathrm{HPBW}}^{xz}\right)\;,$$

where $\bar{z}$ is the range distance between the aperture and the object’s center and $\theta_{\mathrm{HPBW}}^{xz}$ is the antenna’s half-power beamwidth in the xz plane. This lateral aperture is then divided into equal sub-areas of extent on the order of one to ten $\lambda_{c}$, where $\lambda_{c}$ is the wavelength at the central frequency of the band of operation.

The random sampling strategy must provide coverage of the entire lateral domain. This is why we impose the requirement to collect at least one sample with lateral coordinates within each sub-area before performing the first image-convergence check. This ensures that each sub-area is adequately represented in the dataset, preventing regional bias. Similarly, each subsequent convergence check is performed only after at least one additional sample is acquired within each sub-area.

We emphasize that the convergence check is applied only to the OUT power map $M(x^{\prime },y^{\prime },z^{\prime })$ in (6), which serves as an input to the second SPM reconstruction step. The second reconstruction step is not performed until a convergent OUT map emerges. The convergence check is implemented with an SSIM threshold of 0.97 as

(15) $$\mathrm{SSIM}\left(|{\bar{M}}_{p}\left(r^{\prime }\right)\left|,\right|{\bar{M}}_{p-1}\left(r^{\prime }\right)|\right)\geq 0.97\;,p=N_{p},N_{p}-1,N_{p}-2,N_{p}-3\;,$$

where $|{\bar{M}}_{p}\left(r^{\prime }\right)|$ is the normalized absolute value OUT power map in the *p*-th convergence check and $N_{p}$ is the total number of convergence checks reached. It is clear from (15) that we employ four consecutive SSIM image comparisons, all of which must exceed the threshold. This is necessary because, with random sampling, the image convergence is not smooth or monotonic. Once the SSIM check satisfies (15), the data acquisition is terminated and the algorithm proceeds to the second stage of the SPM method to perform image enhancement and quantitative image reconstruction (with measured PSFs).

We briefly note that with large sub-areas (a size of $3\lambda_{c}$ or more), it may be computationally prudent to impose a minimum number of collected samples to start the image-convergence checks. It is unlikely that image convergence will start unless there is at least one sample within an area of $\lambda_{c}\times \lambda_{c}$ on average.

**Figure 2:**
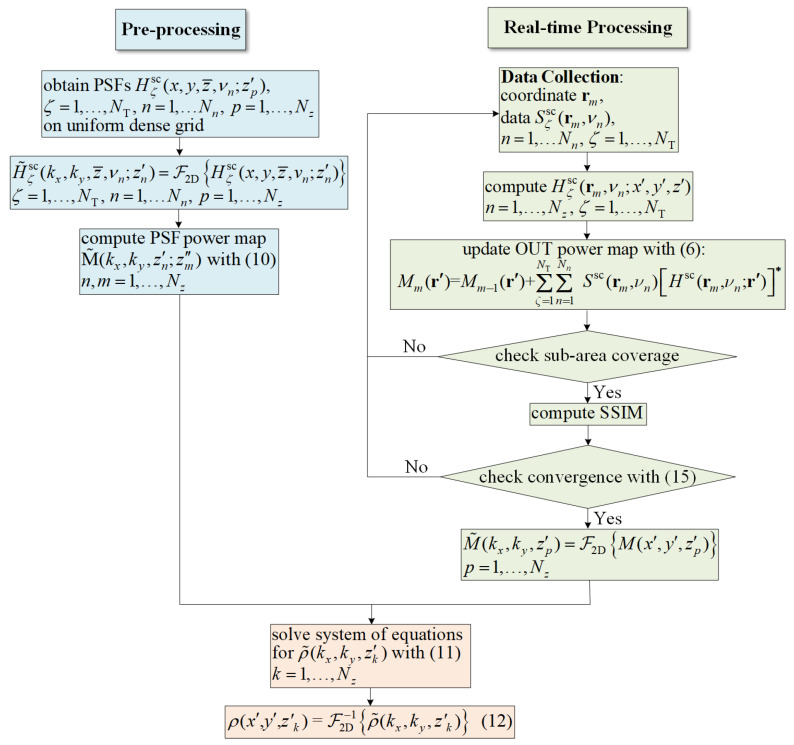
Flowchart of the SPM algorithm for random sampling.

The proposed random sampling algorithm is summarized in Figure 2. The green and blue blocks represent the implementation of the first stage of SPM, while the orange blocks correspond to the second stage. The flowchart is divided into two main sections: the blue arm and the green arm. Before initiating the random sampling procedure, the formation of the SP power maps is necessary, which is shown in the blue blocks of Figure 2. After both SP and OUT power maps are obtained in the Fourier domain, the algorithm proceeds to the second stage of SPM to acquire quantitative estimates of permittivity.

## 3. Validation Examples with Simulated Data

To validate the efficacy of the SPM algorithm in reconstructing the permittivity of objects from random spatial samples, we carry out experiments with data obtained from simulations with the Altair FEKO full-wave simulator [93] and an in-house LFM radar simulator [78] developed in MATLAB [94].

In general, the image reconstruction needs to de-embed the background radar responses from the total-field responses. The background responses are those acquired in the absence of an object under test (OUT), whereas the total-field responses are acquired in the presence of the OUT. The FEKO simulations employ realistic dipole antennas in both monostatic and bi-static measurement setups. Thus, the background responses (reflection and transmission *S*-parameters) are significant and need to be acquired and de-embedded. Since the background medium is assumed homogeneous, only one simulation is needed, where the antennas transmit/receive in the employed antenna-array configuration without an OUT. The de-embedding employs a simple subtraction of the background responses from their OUT counterparts to obtain the needed scattered field responses. On the other hand, the LFM radar simulator generates the scattered field responses directly employing an analytical scattering model [78]. Therefore, the LFM radar examples do not require a background response acquisition.

Further, the system PSFs $H_{\zeta }^{\mathrm{sc}}\left(r,\nu ;r^{\prime }\right)$, ν=ω,t, are needed in the first SPM reconstruction stage to compute the OUT power map (6) using the randomly sampled OUT data. The LFM simulator computes those analytically using the distances $R_{\mathrm{Tx}}\left(r,r^{\prime }\right)$ and $R_{\mathrm{Rx}}\left(r,r^{\prime }\right)$ between the scattering probe at $r^{\prime }$ and the Tx and Rx antennas, respectively [78]. In our notations, the Rx antenna position is r whereas the Tx antenna position is determined from r since the antennas are in a fixed array configuration. On the other hand, the PSF acquisition with FEKO simulations involves a simulated measurement scan of an electrically small scattering probe (SP) positioned at the center of an imaged slice $\left(0,0,z^{\prime }\right)$. Each imaged slice ($z_{p}^{\prime }$, $p=1,2,\ldots ,N_{z}$) requires a simulated scan to acquire $H_{\zeta }^{\mathrm{sc}}\left(x,y,\bar{z},\omega ;0,0,z_{p}^{\prime }\right)$. Here, $\bar{z}$ is the range position of the synthetic aperture where the respective Rx antenna scans. The PSFs for any other lateral position of the SP $\left(x^{\prime },y^{\prime },z_{p}^{\prime }\right)$ are obtained by a coordinate shift, i.e., $H_{\zeta }^{\mathrm{sc}}\left(x,y,\bar{z},\omega ;x^{\prime },y^{\prime },z_{p}^{\prime }\right)=H_{\zeta }^{\mathrm{sc}}\left(x-x^{\prime },y-y^{\prime },\bar{z},\omega ;0,0,z_{p}^{\prime }\right)$.

Additionally, as explained in Section 2, the *k*-space PSFs ${\tilde{H}}_{\zeta }^{\mathrm{sc}}\left(k_{x},k_{y},\bar{z},\nu ;z_{p}^{\prime }\right)$, $p=1,2,\ldots ,N_{z}$, are needed to compute the *k*-space PSF power maps $\tilde{M}\left(k_{x},k_{y},z^{\prime };z^{\prime \prime }\right)$ using (10). We reiterate that this computation employs the 2D FTs of the real-space PSFs $H_{\zeta }^{\mathrm{sc}}\left(x,y,\bar{z},\nu ;z_{p}^{\prime }\right)$, $p=1,2,\ldots ,N_{z}$, acquired on a uniformly sampled “dense” 2D grid on a planar synthetic aperture $S_{a}$, where the spatial sampling steps Δx and Δy conform to the Nyquist limit of $\frac{\lambda_{\min}}{4\sin\alpha }$. These steps dictate the pixel size of the image. The PSF power maps are independent of the OUT and are precomputed.

To emulate an OUT measurement with random spatial sampling, we first define a uniform dense-grid observation domain, where $\Delta x=\Delta y\approx \frac{\lambda_{c}}{4}$. We perform simulations with the OUT and acquire the OUT responses at all observation points in this grid. Subsequently, an algorithm randomly selects the points whose responses are utilized to compute the OUT power map using (6). As detailed in Section 2.3, we divide the observation domain into equal sub-regions. In all examples, we employ 16 sub-regions, the lateral extent of which does not exceed $10\lambda_{c}$.

**Figure 3:**
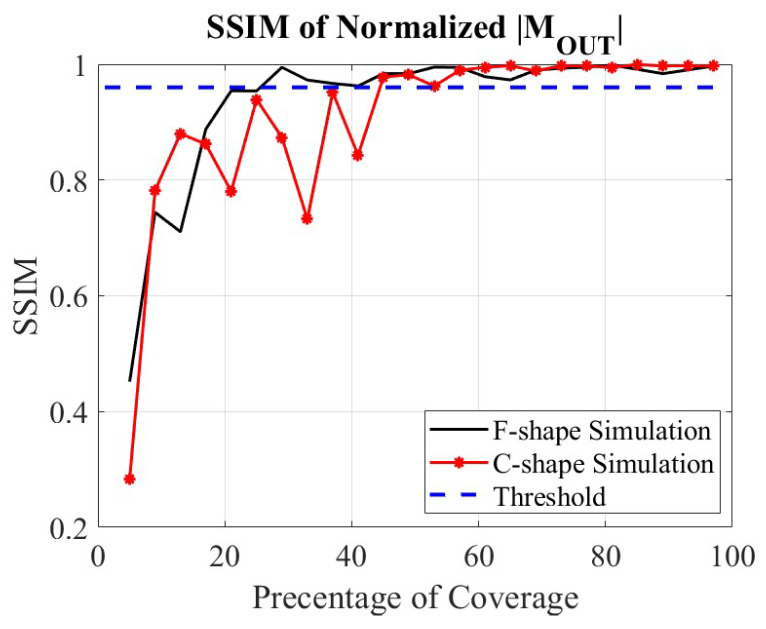
Image convergence in the F shape and the C shape simulated examples in terms of the SSIM between two subsequent OUT power maps *versus* the amount of used spatial samples (in percentage) relative to the number of samples in the dense (Nyquist-compliant) grid on the planar synthetic aperture $S_{a}$.

After collecting the minimum required number of points (at least one point in each designated sub-region), the generation of the first OUT power map $M_{1}\left(r^{\prime }\right)$ is complete, and this map is stored to be compared with the subsequent updated power map $M_{2}\left(r^{\prime }\right)$. The process continues until image convergence is observed as per (15). As an illustration, Figure 3 shows a plot of the SSIM value versus the amount of used spatial samples (in percentage) relative to the number of samples in the dense (Nyquist-compliant) grid on the planar synthetic aperture $S_{a}$. It is observed that in both simulation examples, the OUT power maps converge with about 50% of the sample point, which would be required by a uniform sampling scan.

### 3.1. C Shape Image Reconstruction with Simulated Data

**Figure 4:**
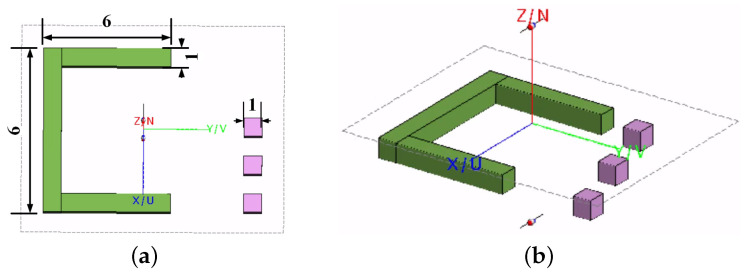
The full-wave FEKO simulation example of a 3D structure: (**a**) top view and (**b**) isometric view. The C shape and the three small cubes reside in three range slices ($z^{\prime }=3$ cm, $z^{\prime }=4$ cm, and $z^{\prime }=5$ cm). Two dipoles are positioned 8 cm apart so that the C shape is centered between them. The C shape is 7×9×1 cm^3^, and the cubes are all 1 cm^3^. The C shape and the cubes have relative permittivity of $\varepsilon_{r}=1.5$ and $\varepsilon_{r}=1.1$, respectively. Background is vacuum, $\varepsilon_{r,b}=1$. All dimensions are in cm.

In the FEKO simulation, a scenario similar to that in previous studies is replicated [95]. The OUT consists of a C-shaped dielectric object in the $z^{\prime }=0$ plane with a relative permittivity of $\epsilon_{r}=1.5$. Adjacent to it, three small cubes with a permittivity of $\epsilon_{r}=1.1$ reside at distances of 3 cm, 4 cm, and 5 cm along the *z* axis and with a slight offset (center-to-center distance of 2 cm) along the *y* axis. The background is a vacuum ($\epsilon_{r}=1$). The setup is depicted in Figure 4.

The PSFs are acquired by simulating the scans of a scattering probe (SP) at the center $\left(x^{\prime }=y^{\prime }=0\right)$ of three imaged planes: $z^{\prime }=3,4,5$ cm. The SP is a small cube, 1 cm on a side, with a relative permittivity $\varepsilon_{r}=1.1$.

The measurement setup involves two half-wavelength (at the central frequency of 5 GHz) dipole antennas spaced 8 cm apart, with the OUT centrally located between them. The imaged domain contains slices that are as close as 3 cm from the antennas. Although we are at the object’s close range, this is still a borderline far-field scenario with respect to the antenna’s lateral extent. The two antennas collect reflection, $S_{11}$ and $S_{22}$, as well as transmission, $S_{21}$, data across a frequency range from 3 GHz to 8 GHz and over a 30 cm by 30 cm acquisition plane. We divide this aperture into 16 equal sub-regions, the lateral extent of which is about $1.4\lambda_{c}$. On the other hand, the sampling interval employed in acquiring the dense uniform grid PSFs is $0.18\lambda_{c}=1$ cm. Note that with monostatic measurements such as $S_{11}$ and $S_{22}$, the Nyquist criterion dictates the sampling interval from a quarter-wavelength to half-wavelength, depending on the maximum viewing angle of the antenna beam and the aperture size [38]. The dense uniform grid sampling is also utilized in the OUT simulations, which provides a high-quality image, serving as a benchmark to assess the images reconstructed from the randomly sampled data.

**Figure 5:**
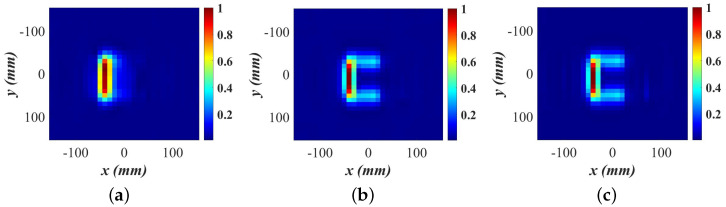
The slice at $z^{\prime }=0$ of the normalized magnitude OUT map $\bar{M}\left(r^{\prime }\right)$ of the C shape generated by the first SPM stage for percentage of utilized spatial samples selected randomly: (**a**) 40%, (**b**) 70%, and (**c**) 100%. The total number of spatial samples in the dense uniform sampling grid is 961.

Figure 5 shows the slice at $z^{\prime }=0$ of the normalized magnitude OUT map $\bar{M}\left(r^{\prime }\right)$ of the C shape generated by the first SPM stage as the map evolves while the spatial samples are collected randomly. The reported percentages reflect the proportion of samples used relative to the total available samples (961) acquired on the dense uniform grid.

Figure 5a shows the image obtained with 40% of the entire dataset, corresponding to only 385 randomly selected spatial samples. It captures only the vertical arm of the C shape, and the small cube in this slice is not visible due to the fact that the horizontal arms are not covered as well as the vertical arm at the early stages of the random acquisition. Consequently, their contribution to the formed image is less pronounced. As the scanning progresses, with additional samples collected in each sub-region, the complete C shape starts to emerge. By the time 70% of the area sample points are utilized and the convergence is met according to Figure 3, the C shape is clearly formed, although the small cube is still not visible as depicted in Figure 5b. The scattering from this low-contrast small cube is weak, and even with 100% of the dense-grid samples, the OUT power map does not depict this detail well; see Figure 5c.

**Figure 6:**
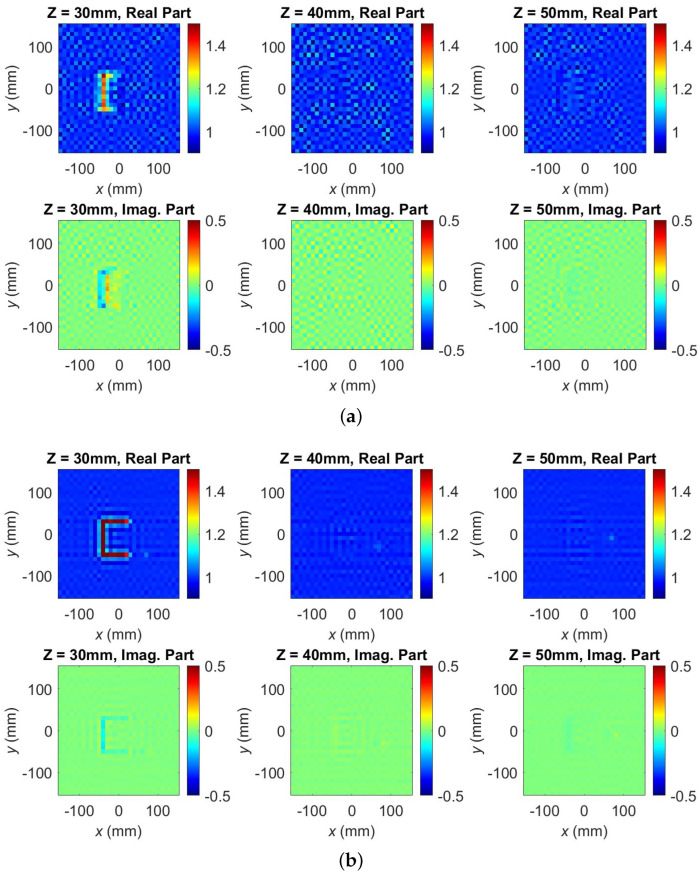
Quantitative SPM reconstructed images in terms of real and imaginary parts of the C shape relative permittivity utilizing randomly sampled data of the object shown in Figure 4 with (**a**) 40% and (**b**) 70% of the dense-grid samples. The images in each column correspond to a range slice.

The small cubes in all three image slices become visible only after the second SPM stage. At 70% utilization of the dense-grid points, the SSIM value is 0.988, and the C shape power map is already convergent; see also Figure 3. At this stage, the scan is terminated. The three slices of the OUT power map are subjected to 2D FT and the OUT reflectivity is recovered by the second SPM stage; see (11). The recovered quantitative complex permittivity images are of much better structural and resolution quality than the OUT power map. Figure 6a illustrates the quantitative C shape image slices formed using 40% of the dense dataset for the OUT power map, resulting in an SSIM value of 0.86, indicating incomplete recovery of the C shape. The converged result, shown in Figure 6b, utilizes 70% of the dense dataset, demonstrating a marginal improvement post-convergence and a 30% reduction in required measurements compared to the Nyquist-compliant dense-grid scan (100% data). The SSIM value between the 100% and 70% reconstructed images is 0.999.

### 3.2. F Shape Image Reconstruction with LFM Radar Synthetic Data 

The in-house LFM radar simulator employs a far-zone scalar scattering model, which assumes point-like sources (the term *point source* refers to a scalar-field source whose radiated field is represented by $e^{-jkr}/r$ (a spherical wave). The spherical wave represents adequately the distance dependence of antennas’ far-zone fields [96]). It accounts for the signal decay due to the spherical spread of the transmitted and scattered waves. However, it ignores the depolarization that may occur upon scattering. With these assumptions, the LFM system PSF is analytically expressed as [78]

(16) $$H^{\mathrm{sc}}\left(r_{m},t;r^{\prime }\right)=\frac{A_{\mathrm{Tx}}}{R_{\mathrm{Tx},m}R_{\mathrm{Rx},m}}P\left(\frac{t-\tau_{d,m}}{T_{p}}\right)\exp\left[-i2\pi \left(f_{c}+\gamma t\right)\tau_{d,m}\right]\;,$$

where $A_{\mathrm{Tx}}$ is the amplitude, $f_{c}$ is the center frequency, *t* is the fast time (the time within a single chirp), $T_{p}$ is the chirp duration (pulse width), $\gamma =\frac{B}{T_{p}}$ is the frequency-modulation slope (the chirp rate), and *B* is the chirp’s frequency bandwidth. $R_{\mathrm{Tx},m}=\left|r_{\mathrm{Tx},m}-r^{\prime }\right|$ and $R_{\mathrm{Rx},m}=\left|r_{m}-r^{\prime }\right|$ are the distances from the Tx antenna at $r_{\mathrm{Tx},m}$ and the Rx antenna at $r_{m}$ to the scattering point at $r^{\prime }$, respectively. Note that the range compression in LFM radar signals is already taken into account in the derivation of the analytical PSF in (16), where the correlation of the received signal with the reference (transmitted) signal produces the down-converted (baseband) signal. We reiterate that the Tx and Rx antenna pairs are in a fixed configuration; therefore, $r_{\mathrm{Tx},m}$ is determined from $r_{m}$. Further, $\tau_{d,m}$ is the time delay corresponding to the distance traveled by the signal, i.e., $\tau_{d,m}=\left(R_{\mathrm{Tx},m}+R_{\mathrm{Rx},m}\right)/c$, where *c* is the speed of light. Note that here we employ point sources and point sampling and the scenario is a far-field one. As shown in (3), the linearized forward model of scattering views the signal from an object as a superposition of the scattering emanating from all differential scatterers that make up this object. Thus, using (16), the cumulative OUT signal is synthesized using the time-domain counterpart of the superposition integral in (3).

In the synthetic experiment, the LFM imaging-system parameters are first set. Here, we present a monostatic case, i.e., the Tx and Rx positions are coincident. The central frequency $f_{c}$ is 29.9 GHz, while the covering frequencies are from 27.0 GHz to 32.8 GHz. The chirp duration $T_{p}$ is 20 μs and the chirp rate is $\gamma =2.9\times 10^{14}$ Hz/s. Overall, there are 201 time samples in a single chirp. The lateral spatial resolution is estimated from the wavelength at $f_{c}$ as $\delta_{⊥}\approx \lambda_{c}/4\approx 2.5$ mm and the range resolution is $\delta_{z}\approx c/2B\approx 26$ mm.

The reference SPM image is obtained from OUT and PSF scans on a uniform grid over a planar aperture of size 200×200 mm^2^, with spatial increments of 4 mm along *x* and *y*. The aperture is at $\bar{z}=100$ mm. This results in a dense grid of 51×51 (2601) sampling points. The PSF is acquired with a cubical SP of size 1 mm^3^ and $\varepsilon_{r}=1.5$. We reiterate that this PSF is needed not only to obtain the reference OUT power map but also to carry out the second SPM stage, regardless of whether the OUT data are acquired on a random trajectory or on the dense uniform grid planar aperture.

**Figure 7:**
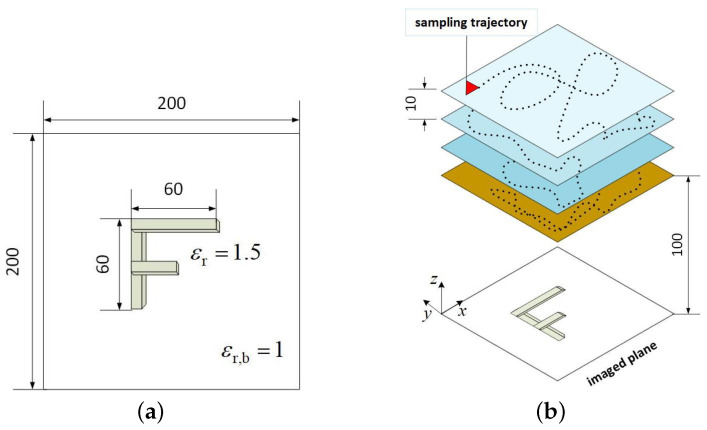
Illustration of a monostatic LFM radar simulation example: (**a**) the F shape object of relative permittivity $\varepsilon_{r}=1.5$ at $z^{\prime }=0$, and (**b**) 3D sampling trajectory in four planes at $\bar{z}$ = 100, 110, 120, and 130 mm. Background is vacuum, $\varepsilon_{r,b}=1$. All dimensions are in mm.

The random scanning of an F shape object is implemented in the LFM radar simulator as shown in Figure 7. The F shape, of relative permittivity $\varepsilon_{r}=1.5$, is built of cubical scatterers 1 mm on a side in the $z^{\prime }=0$ plane; see Figure 7a. The background is a vacuum ($\varepsilon_{r,b}=1$). The F shape has vertical and horizontal arms of length 60 mm whereas the middle arm measures 30 mm. To emulate 3D random sampling, four planes along *z* are set so that they are 10 mm apart ($\bar{z}$ = 100, 110, 120, and 130 mm). The sampling trajectory for the OUT measurement follows a random selection along *x*, *y*, and *z* as shown in Figure 7b.

The methodology is the same as in the C shape example. The 3D (four-slice) sampling domain of overall lateral extent 200×200 mm^2^ is divided into 16 equal 3D sub-regions of lateral extent 50×50 mm^2^. As before, the convergence check on the OUT image is performed only if each additional dataset provides at least one sample within each sub-region. Remarkably, the OUT map converges with a number of 3D random samples which constitutes only 40% of the total sample number on the dense planar grid (2601); see the SSIM plot in Figure 3.

**Figure 8:**
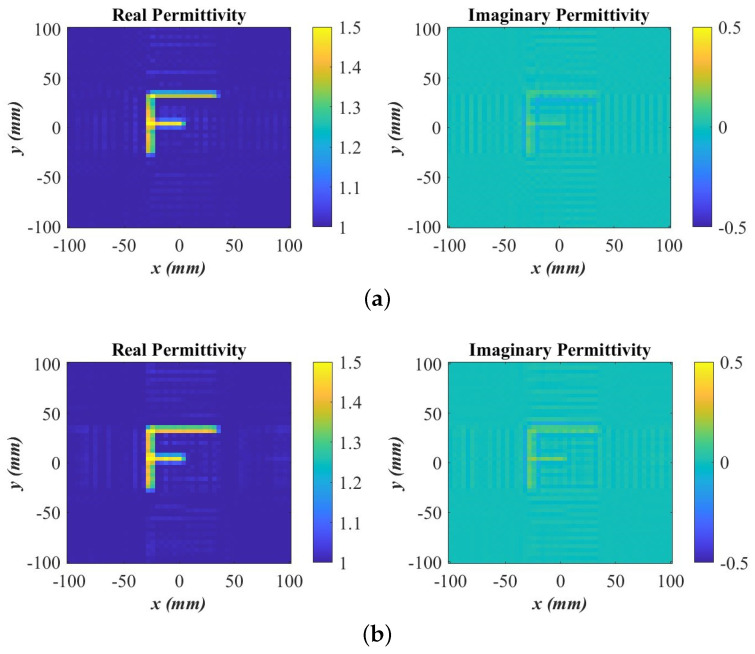
Quantitative reconstructed images in terms of real and imaginary parts of the relative permittivity of the F shape object utilizing randomly sampled data: (**a**) using the convergent OUT map obtained from random samples, the number of which is only 40% of the number of samples on the dense uniformly sampled planar grid and (**b**) using all the samples on the dense uniformly sampled planar grid.

For brevity, we only present the final quantitative (second-stage) image in Figure 8a obtained with a number of random samples amounting to 40% of the sample number of the dense planar synthetic aperture. As a reference, the quantitative image obtained with the full dataset acquired on the dense uniformly sampled planar aperture is shown in Figure 8b. We again observe only marginal improvement in comparison with the image obtained from the under-sampled random data while reducing the required measurements by 60%. This illustrates the importance of employing the image convergence of the OUT map to terminate the measurements with random sampling in a timely manner. In conjunction with the previous C shape example, it also shows that the number of required random samples can vary significantly depending on the employed radar system (continuous wave versus LFM) as well as the complexity of the imaged scene.

## 4. Validation Examples with Measured Data

The experiments discussed here are first carried out using a uniform grid in a planar raster-scanning chamber. These measurements provide a full OUT dataset, from which samples can be selected randomly.

The PSFs are acquired experimentally in the near-field imaging experiment (breast phantom scan), presented in Section 4.1. Near-field measurements allow for capturing the scattered responses from an electrically small SP in the background medium. This is due to the short distance between the synthetic aperture and the SP. Since the SP is small, its scattering is weak; however, the antennas are very close and the captured signals provide a sufficient signal-to-noise ratio (SNR). With measured PSFs, the SPM algorithm reconstructs quantitative images.

On the other hand, in the far-field experiments with mm-wave LFM radar, presented in Section 4.2, measuring the system PSF is not possible. The scattering from an electrically small SP is so weak that it does not provide a sufficient SNR when the SP resides in the antenna’s far zone. Thus, in the far-zone experiments, we employ the same analytical PSFs as in the simulation-based example presented in Section 3.2. In such far-field measurement scenarios, there is no significant difference between the OUT power map (SPM first-stage image) and the second-stage image. Importantly, since the analytical PSF is incapable of accounting for setup specifics such as antenna patterns, cable losses, and illumination strength, the second-stage SPM images are only qualitative. Consequently, the OUT permittivity distribution is simply presented in terms of the normalized reflectivity.

**Figure 9:**
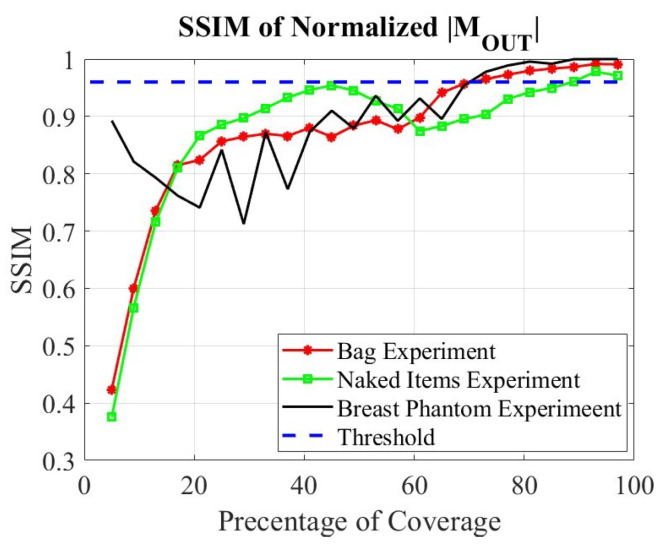
Convergence curves for the experimental examples in terms of SSIM between two subsequent OUT power maps versus number of randomly selected samples (in percentage) relative to the total number of samples on the dense uniform grid on the synthetic aperture.

Figure 9 illustrates the SSIM curves for each conducted experiment. As in the examples with simulated data, the scan-termination threshold is at SSIM=0.97. As demonstrated next, this rigorous convergence criterion ensures that satisfactory image quality is achieved before terminating the scan.

### 4.1. Compressed Breast Phantom Imaging

**Figure 10:**
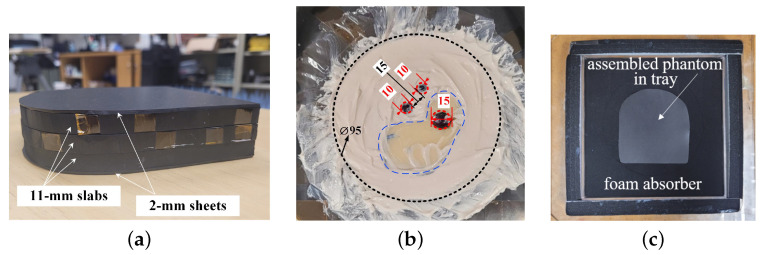
Photos of compressed breast phantom: (**a**) side view showing the three carbon–rubber slabs with permittivity close to that healthy breast tissue and the two thin silicone–rubber sheets as skin simulant, (**b**) middle layer including two tumor simulants surrounded by the matching material and one surrounded by the fibroglandular simulant, and (**c**) assembled phantom surrounded by microwave-absorbing foam. All dimensions are in mm.

A near-field imaging experiment identical to the one presented in [97] is carried out to evaluate the effectiveness of the SPM method with randomly sampled data. The scanned aperture measures 18×18 cm^2^. The dense aperture scan utilizes an interval of a 3 mm (approximately $0.05\lambda_{c}$) axis, creating a grid of 61×61 spatial samples. Consistent with other examples, the domain is divided into 16 sub-regions, each measuring $4.5\times 4.5\;{\mathrm{cm}}^{2}$ (approximately $1.3\lambda_{c}$). The measurements are performed on a heterogeneous compressed breast phantom, which includes three tumor simulants immersed in various healthy tissue simulants. The breast phantom (the OUT) is made of three layers of 11 mm-thick carbon–rubber slabs mimicking the electrical properties of healthy scattered fibroglandular breast tissue and two 2 mm-thick silicone–rubber sheets mimicking skin tissue (see Figure 10a). The 2 mm-thick silicone–rubber sheets are placed on the top and the bottom of the three stacked 11 mm-thick carbon–rubber slabs. The average relative permittivity of the slabs (listed in Table 1) is chosen to match the averaged complex permittivity of BIRADS Type II density of the breast (scattered fibroglandular tissue). The breast phantom also comprises a number of dispersed materials, as shown in Figure 10b. Each slab defines a layer where additional tissue simulants can be inserted. In this phantom, Layers 1 and 3 are homogeneous. In Layer 2, a circular section (a 94 mm diameter) is removed to insert various tissue simulants. A kidney-shaped material mimicking the healthy fibroglandular tissue (see Figure 10a) is put inside the circular section. One tumor simulant is inserted within this fibroglandular object and two tumor simulants are embedded in the circular section carefully surrounded by a matching medium with a relative permittivity reported in Table 1. The phantom is placed in a *Plexiglass* tray and surrounded by black foam microwave absorbers, as shown in Figure 10c, to minimize reflections and to reduce the image artifacts.

The setup for measuring the system PSF is the same as the OUT measurement setup except for the second layer where a small scattering probe (a cylinder of height 1 cm and diameter 4 mm) with the relative permittivity value given in Table 1 is positioned at the center of an otherwise homogeneous carbon–rubber slab. Finally, in order to extract the scattered field responses for both the OUT and the PSF measurements, a background measurement is necessary. The background object is composed of three homogeneous carbon–rubber slabs with the two silicone–rubber sheets at the top and the bottom, surrounded by microwave-absorbing foam. All these measurements inherently incorporate the properties of the actual antennas.

**Figure 11:**
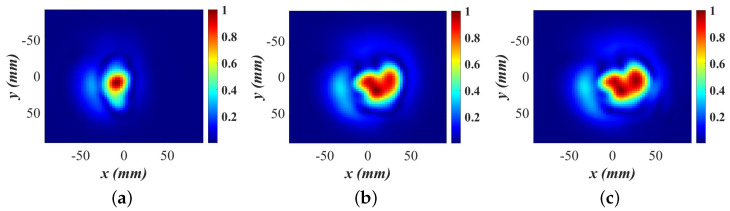
Normalized OUT power maps of the compressed breast phantom, when the number of randomly selected samples (in percentage) relative to the total number of samples on the planar synthetic aperture is (**a**) 50%, (**b**) 70%, and (**c**) 100%. The total number of spatial samples is 3721.

Figure 11 shows the normalized OUT power maps of the compressed breast phantom obtained from randomly sampled data at three percentage values of the utilization of the available densely sampled data. The reconstructed relative permittivity distribution is represented as 2D projections since the imaging setup uses transmission coefficients only. Note that the Rx array elements are at the bore–sight (or only slightly off) of the Tx antenna, which results in the lack of range resolution. Figure 11a shows the result when processing only 50% of the points on the dense (Nyquist-compliant) grid. At this stage, the SSIM is well below the threshold indicating insufficient image quality; see Figure 9. Indeed, the comparison of the image in Figure 11a with those in Figure 11b (70% utilization of the points on the dense grid) and Figure 11c (100% utilization) confirms incomplete OUT map reconstruction. The OUT maps in Figure 11b,c are visually similar, but the third tumor simulant on the right side of the fibroglandular region is not discernible. Note that the SSIM value at the 70% stage reaches 0.97; see Figure 9. However, as discussed in (15), the convergence criterion requires the SSIM to be greater than the threshold in four consecutive iterations. For this reason, the scan continues for another three batches of sampled data. The data eventually converge when 80% of the reference data are utilized and they are then terminated to proceed to the second stage of the SPM algorithm.

We briefly mention the importance of using an apodization filter [98] in this challenging experimental example. The challenges in the image reconstruction here arise from the significant reflections arising at the interface between the breast phantom and the microwave foam. The apodization filter is aligned with the boundary of the circular section in Layer 2; see the black dashed line in Figure 10b. Here, a 2D Butterworth apodization filter is used, which is applied radially over a circular region. The cut-off −3 dB level corresponds to a radial distance of about 4 cm from the center. The apodization filter suppresses the contribution of the samples outside the circular boundary, thus mitigating reflections due to imperfect absorption by the microwave foam.

**Figure 12:**
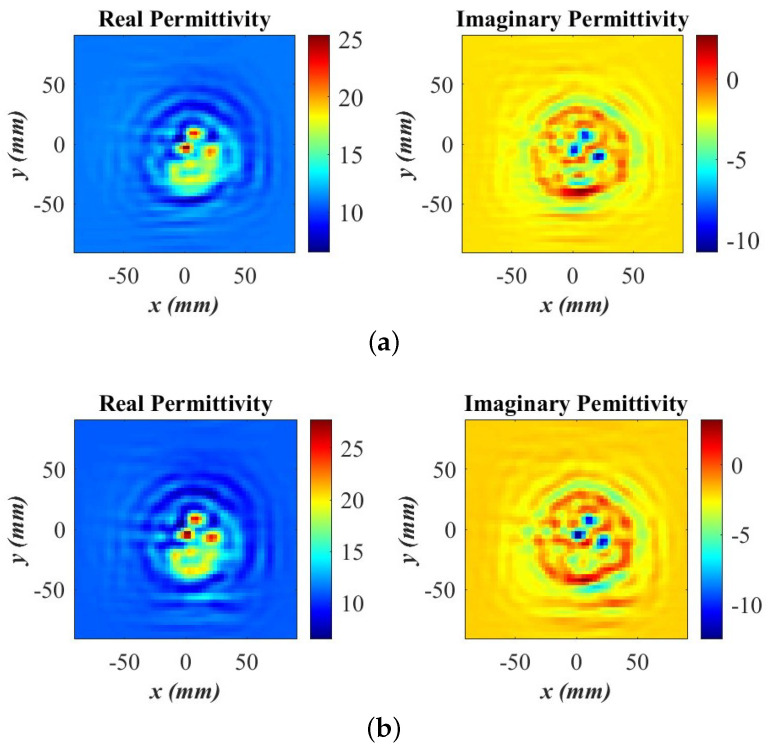
Quantitative SPM reconstructed images in terms of real and imaginary parts of the breast phantom permittivity utilizing randomly sampled data in percentage proportion of all available samples: (**a**) 70% and (**b**) 80%.

The quantitative reconstruction of the breast phantom relative permittivity by the SPM second stage is shown in Figure 12. Figure 12a shows the reconstruction result with the OUT power map based on 70% of all available samples, selected randomly, whereas Figure 12b shows the quantitative image when using the OUT power map which employs 80% of all available samples with the SSIM of 0.99 compared to the reference image (100% densely sampled), also selected randomly. In both image sets, the tumor simulants and the healthy fibroglandular tissue regions are reconstructed well, with the tumor simulants correctly identified by large permittivity values (both real and imaginary). As expected, the converged image utilizing the 80% set of available data features slightly better spatial resolution, which results in a better structural outline of the phantom inclusions. The permittivity value distributions of the 2D images in Figure 12 are effectively averaged over the thickness (3.7 cm) of the phantom. Thus, the values are lower than those provided in Table 1.

We briefly comment on the benefit of employing filtering at the second SPM stage. Low-pass filtering is applied to the reconstructed complex permittivity in *k*-space [98] before applying the inverse 2D FT. This is important in suppressing image artifacts when experimental data are used. High spatial (*k*-space) frequency components correspond to near-grazing angles of signal arrival and, in near-field imaging, evanescent-field scattering. These components suffer from a very poor SNR, which may corrupt the final reconstruction.

Finally, it is noteworthy that the reconstruction algorithm operates with remarkable speed during the relative permittivity retrieval, typically completing in a few seconds on conventional laptops using MATLAB codes without code optimization, acceleration, or parallel computing. The enhanced imaging results after applying the second SPM stage, as evidenced by the comparison of the images in Figure 12 with those in Figure 11, underline the importance of this stage.

### 4.2. Imaging of Various Small Items with mm-Wave LFM Radar

The off-the-shelf radar module used for LFM radar measurement is the IWR1443Boost evaluation module [99] along with the real-time data-capture adapter board DCA1000EVM [100]. The mm-wave sensor is equipped with three Tx and four Rx antennas; however, in this experiment, only one Tx is activated while all four Rx elements receive. Here, too, we are at the far-field of the antennas. The LFM transceivers can accommodate up to a 4 GHz bandwidth from 77 GHz to 81 GHz. The chirp duration is $T_{p}=51.1\;\mu$s with 512 temporal samples. The frequency-modulation slope is γ=72.42 MHz/μs. The off-the-shelf mm-wave radar performs the range compression on hardware (with mixing) and provides as an output only the baseband in-phase (I) and quadrature (Q) outputs as a function of time.

**Figure 13:**
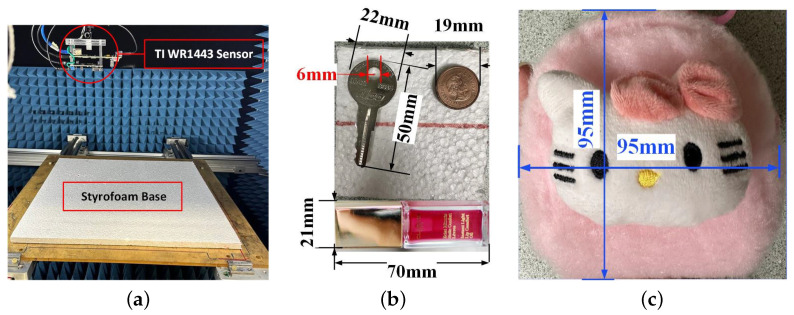
Photos of (**a**) the acquisition chamber; (**b**) a key, a penny, and a liquid lipstick laid on a Styrofoam sheet; and (**c**) a toy bag where the key, penny, and lipstick are inserted in the first imaging experiment.

The imaging setup and the measured objects repeat an experiment reported in [78] (see Figure 13a). The scanned aperture has a size of 15×15 cm^2^. The dense aperture scan employs an interval of 2 mm (approximately $0.53\lambda_{c}$) along *x* and *y*, resulting in a grid of 76×76 spatial samples. As per other examples, the domain is divided into 16 sub-regions, each with a size of $3.75\times 3.75\;{\mathrm{cm}}^{2}$ (approximately $10\lambda_{c}$).

It is important to note that the LFM radar module suffers from internal system delays; therefore, the analytical PSF needs to be calibrated. Here, we use the calibration approach described in [78]. Further, in the LFM radar experiments presented here, background de-embedding is not necessary since the background signals are negligible compared to the back-scattering from the objects. Also, the system PSF is computed analytically using (16). Therefore, background and PSF measurements are unnecessary.

In the first imaging experiment, the OUT consists of a metallic key, a penny, and a liquid lipstick (see Figure 13b), enclosed within a toy bag, shown in Figure 13c. All three objects are laid on a Styrofoam sheet and then inserted in the bag. The OUT is 22.5 cm away from the radar. In the second experiment, the same objects are imaged without the bag and at the same range distance.

**Figure 14:**
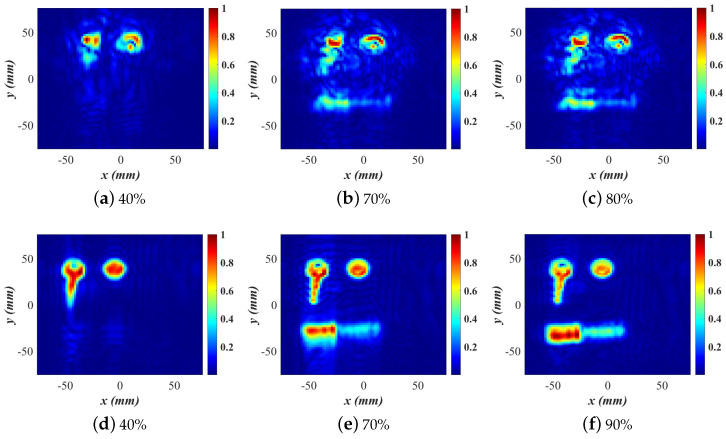
Qualitative results in terms of normalized magnitude of reflectivity $\bar{\rho }$ distribution for (**a**–**c**) the embedded objects in the bag and (**d**–**f**) naked objects. The percentage of random samples compared to the total available uniformly sampled data is 40%, 70%, 80%, and 90% as denoted in the figure.

Figure 14a–c show 2D images of the key, penny, and lipstick experiment, concealed in the bag, at various stages of completion of the random sampling and when the convergence is at an SSIM of 0.98 when 80% of the referenced samples are utilized. The images are obtained at a single slice at z=21.5 cm from the radar antenna array. The 2D images of the same objects, this time out of the bag, are depicted in Figure 14d–f. Here, the convergence is when 90% of the densely sampled reference data are used and the scan is terminated at the SSIM of 0.99. It is clear that the bag obscures the objects to some extent, which is expected since the materials from which the bag is made are not entirely transparent to the mm-wave radiation. Note that the images in Figure 14a–c indicate the presence of the bag by a low-reflectivity region surrounding the objects.

The adequacy of the selected points is demonstrated through the experiments depicted in Figure 14c,f, corresponding to the bagged and unobscured conditions, respectively, which indicates where the scan can be terminated. In both scenarios, the visual representation of the key and the penny closely matches those observed with 100% random sampling. Therefore, the 100% data are not presented for brevity. The impact of the insufficient data (40%) is particularly evident in Figure 14d, where the lipstick is not detectable at all and the key’s leg is also not recovered well. The SSIM value of 0.92 also indicates that more acquisition points are required to reach convergence.

In the bag experiment, utilizing 40% of the data results in an SSIM value of 0.88, which falls below the established threshold, as shown in Figure 9. When data coverage reaches approximately 70%, the SSIM value meets the 0.97 threshold. Subsequently, as the SSIM value remains above this threshold for the next three measurements, the scanning process is discontinued thereafter at 80% of coverage. Conversely, in the experiment with unbagged items, convergence is achieved only after nearly 90% of the data are utilized. Nonetheless, the image resolution at 70% coverage is deemed adequate, as illustrated in Figure 14e.

## 5. Discussion

To demonstrate the advantages of our SPM method in the context of nonuniform random sampling, we should consider multiple factors when comparing it with the conventional BPA and ω-*k* methods.

First, we analyze the overall computational complexity, which includes all $N_{m}$ spatial samples acquired during the scan. According to (6), the first SPM stage involves $N_{m}N_{R}N_{n}N_{v}$ multiplications and the same number of summations, where $N_{v}=N_{x}N_{y}N_{z}$ is the number of image voxels. Thus, its computational complexity can be expressed as $O(N_{m}N_{R}N_{n}N_{v})$, which is the same as that of the BPA. The second SPM stage computes the 2D FFT of the OUT power maps (slice by slice) as well as the 2D IFFT of the *k*-space reconstructed permittivity (also slice by slice), both of which have a complexity of $O\left(N_{z}N_{x}N_{y}\log_{2}\left(N_{x}N_{y}\right)\right)\equiv O\left(N_{v}\log_{2}\left(N_{x}N_{y}\right)\right)$. It also solves the system of equations in *k*-space, which has a complexity of $O\left(N_{x}N_{y}N_{z}^{2}\right)\equiv O\left(N_{z}N_{v}\right)$. It is clear that the computational complexity of the second SPM stage is much lower than its first stage. Since the number of range image slices $N_{z}$ is much smaller than $N_{x}$ and $N_{y}$, the computational complexity of the SPM algorithm is governed by the dominant term $O(N_{m}N_{R}N_{n}N_{v})$, which is the same as that of the BPA.

In comparison, the ω-*k* methods, which use NUFFT along with interpolation, have a computational complexity of $O(N_{R}N_{z}N_{x}N_{y}\log_{2}\left(N_{x}N_{y}\right)+N_{n}N_{R}N_{m})$[83,101]. Therefore, if all data are processed after the completion of a scan, the computational complexity of the BPA and the proposed SPM method is higher than that of the ω-*k* algorithms.

Note, however, that the overall computational complexity is not the only factor determining the method’s efficiency in the context of random sampling. The proposed random sampling SPM method ultimately offers significant acceleration over the BPA due to the incremental update of the object’s power map (the first-stage image), performed concurrently with the measurements. It is this incremental image update (with a computational complexity of $O(N_{R}N_{n}N_{v})$) that also allows for employing the proposed image-convergence criterion, which results in a substantial reduction in the number of measurements compared to a Nyquist-compliant uniform densely sampled grid, as demonstrated in the examples. Thus, the proposed method not only leverages computations that run alongside the measurements but also reduces the measurement time. Note that the ω-k methods, just like the BPA, start the image reconstruction only after the scan is completed.

An important advantage of both the SPM and the BPA is their ability to process samples on arbitrary 3D trajectories, noncanonical surfaces, and 3D observation domains. In contrast, the ω*k* approach for nonuniform sampling is limited to sampling on canonical surfaces (planar and cylindrical).

Regarding quantitative analysis, neither the BPA nor the ω-*k* methods can provide quantitative permittivity estimates as they are incapable of utilizing a measured PSF. To the best of our knowledge, the SPM algorithm is the only linear-inversion method that can reconstruct the permittivity distribution of the scanned object. The quantitative imaging is enabled by the second inversion stage of the SPM. Even with analytical PSFs, the second stage of SPM dramatically improves the image quality. Since this stage is absent in the BPA, the BPA image quality is always inferior to that of SPM, even if the SPM method employs analytical PSFs the way the BPA does. The image improvement in the second SPM stage is evidenced by the comparison of the images in Figure 5 and Figure 6 or Figure 11 and Figure 12.

Further, the SPM, just like the BPA, is very effective at suppressing measurement noise, errors, and radar clutter by using a large number of measurements (larger than the number that would be needed in noise-free clutter-free measurements). It is shown in [38] that the SPM first stage minimizes the $\ell_{2}$ norm of the data error, i.e., the difference between the measured responses and the responses that are predicted by the linearized model of scattering with the extracted target permittivity (or reflectivity). $\ell_{2}$ norm solutions are effective in reducing the impact of noise and measurement errors. This is not the case in the ω-*k* algorithms, where the Fourier transforms are sensitive to measurement noise and errors. Note that filtering strategies are also a powerful tool in suppressing radar clutter and rejecting poor SNR data. These have been applied in the example with the compressed breast phantom measurements.

The proposed random sampling SPM method does have limitations, which we address here. One limitation stems from the power map computation in real space, which is significantly slower than the *k*-space computation as shown in [77,78]. However, the *k*-space computation employs 2D FFT, which needs uniformly sampled data and can start only after the scan is completed. The computational complexity of the power map updates with (6) scales with the number of image voxels $N_{v}$. When $N_{v}$∼$10^{6}$ or more, the time required by a sequential computing algorithm may exceed a second, which is slower than the rate at which most imaging radars perform a measurement. For example, off-the-shelf ultra-wideband (UWB) radars can be as fast as 50 waveforms per second [27], but advanced research prototypes may reach rates of ∼1000 measurements per second [102]. To simplify the data-offload management and to reduce the data-storage demands, it is important to achieve a power map update, the speed of which is equal to or better than the speed at which the measurement system provides a spatial sample.

Another limitation in the current investigation is the assumption that the bore–sight axis of the antennas is fixed along the range direction (the *z* axis) and it does not consider a possible tilt of the imaging platform. The platform tilt has two implications: (i) the signal strength depends on the orientation of the antenna beams, and (ii) the signal paths from the Tx antenna to the target and then onto the Rx antenna will change. Both of these implications can be accounted for by the PSFs provided that the antenna-gain patterns and the tilt angle of the platform are known. However, the current implementation does not take these factors into account.

Finally, we emphasize that the proposed method does not employ compressive sensing (CS) strategies; therefore, it is not a solution to the problem of sparse sampling. The reported reduction in the number of spatial samples needed to reconstruct high-quality images is only due to the rigorous convergence criterion, which is automatically determined when the data are sufficient.

## 6. Conclusions and Future Work

This study introduces a novel SPM algorithm optimized for real-time SAR imaging with randomly sampled data on 3D trajectories, a common scenario in mobile and handheld radar platforms. Our approach differs from traditional methods by eliminating the need for uniform sampling across canonical surfaces and spectral estimation techniques, thereby simplifying the computational process and reducing the time requirements. The first stage of the SPM algorithm works concurrently with the measurements on the arbitrary trajectory. With each additional measurement, it updates an evolving scattered power map of the object (a projection image) cast on a uniform 3D grid. The advantage of this updating strategy is three-fold. First, the projection-image update is fast as it amounts to computing and adding $N_{R}\times N_{n}$ terms to the already-existing power map voxel value; see (6). As a reminder, $N_{R}$ is the number of responses measured at each observation position r, and $N_{n}$ is the number of frequency or temporal samples in each response. Second, once the contribution of a spatial sample is added, it is no longer needed, thus eliminating the need to store the entire measurement dataset. This is important in realizing compact mobile or handheld imaging platforms where the image processing is performed on-board and the amount of measurements is extensive. Third, each power map voxel is updated independently, thus allowing for parallel computations with significant acceleration.

The second SPM stage, which is fundamentally an image deconvolution performed in Fourier space, significantly enhances the image quality of the output from the first stage. This stage is computationally very efficient and is independent of the sampling strategy. It, too, is amenable to parallel computations since a small $N_{z}\times N_{z}$ system of Equation (7) is solved independently at each $\left(k_{x},k_{y}\right)$ point in Fourier space. Importantly, the second SPM stage allows for quantitative imaging. We reiterate that quantitative imaging is enabled by measured PSFs, i.e., data obtained by measuring the domain of interest in the presence of a point-like scatterer (not the OUT). When measuring the PSF is not feasible, it is obtained with (2), wherein the incident field distributions of the Tx and Rx antennas are expressed either through analytical formulas or simulated distributions.

A pivotal element of our methodology is the convergence check of the OUT power map update, eliminating the need for a predetermined scan completion. The convergence criterion employs the SSIM between two successive power map updates and an SSIM threshold of 0.97, which must be met by four consecutive SSIM evaluations. It has been observed that the OUT power map converges to a high-resolution image with a number of spatial samples, which is appreciably lower than the number of samples dictated by the Nyquist criterion for a uniformly sampled synthetic aperture. In the imaging of a simple F shape object with simulated data on a 3D trajectory, convergence is achieved with only 40% of the number of samples in the dense uniformly sampled grid on a planar aperture. In addition to the simplicity of the imaged object, the reduction in the spatial samples is likely due to the 3D nature of the observation trajectory, which provides responses at various range distances from the object. On the other hand, in the imaging of a more complex object (key, coin, and lipstick in a bag), measured with an LFM mm-wave radar module, the number of random samples reaches 90% of those on the dense uniform planar grid.

Validation through simulated and measured data confirms the robustness of the proposed approach when applied to both stepped-frequency continuous wave and LFM radar data. The ability to automatically determine the sufficiency of data and to terminate the data acquisition in a timely manner is beneficial in reducing operational costs and time in practical applications.

In order to overcome the limitations discussed in Section 5, upcoming work will focus on implementing the algorithm with parallel programming on multicore, multiprocessor platforms in order to accelerate the computation of the power maps and to achieve real-time processing that matches the speed of the measurements. The second task in the future algorithm enhancement is the ability to take into account the platform tilt by incorporating in the system PSFs the tilt angles and the antenna gain patterns. Third, we aim to enhance the SPM algorithm by integrating it with a compressed sensing (CS) preprocessing step. This integration will enable an OUT power map update directly in *k*-space, achieving convergence with fewer spatial samples, thus accelerating not only the computations but also the measurement process. Finally, we underscore the importance of having accurate measurement coordinates relative to the imaged object. This accuracy is critical for achieving focused images since the spatial resolution is limited to within the accuracy range of the coordinates. Much research and technical solutions are needed in this respect for applications with handheld and mobile platforms. Improving the tolerance of our image-reconstruction method to positioning errors is another focus of future research.

## Figures and Tables

**Table 1:** Averaged dielectric properties of the compressed breast phantom materials over the frequency band from 3 GHz to 8 GHz.

Material (Structure)	$\varepsilon^{\prime }$	$\varepsilon^{\prime \prime }$
Carbon–rubber sheet (averaged breast tissue)	9.6	3.82
Silicone–rubber sheet (averaged skin tissue)	19.36	14
Embedding/matching medium	11.3	2.59
Tumour simulant	64.11	22.32
Fibroglandular tissue simulant	17.61	7.89
Scattering probe (PSF)	43.7	0

## Data Availability

Data are contained within the article.
